# Supplementary material for: A contrast sensitivity model of the human visual system in modern conditions for presenting video content
Source: PLoS One. 2024 May 30;19(5):e0303987. doi: 10.1371/journal.pone.0303987 (PMC11139320; doi:10.1371/journal.pone.0303987)
Supplement: Attachment — Submitted filename: Response to Reviewers.pdf [file pone.0303987.s001.pdf]

Title: A contrast sensitivity model of the human visual system in modern conditions for presenting video content

Authors: Anastasia Mozhaeva, Michael J. Cree, Robert J. Durrant, Igor Vlasuyk, Aleksei Potashnikov, Vladimir Mazin, Lee Streeter

We thank the reviewers for their positive appraisals of our submission, and the editor for the opportunity to revise our work. Below we present the comments and requirements, and our responses in blue. In the manuscript, new and modified text is in red, and text moved in response to the comments and requirements are in blue.

Journal requirements:

1. When submitting your revision, we need you to address these additional requirements. Please ensure that your manuscript meets PLOS ONE's style requirements, including those for file naming.

Thank you for your comment.

We present the manuscript that meets PLOS ONE's style requirements, including those for file naming.

2. We note that your Data Availability Statement is currently as follows: [All relevant data are within the manuscript and its Supporting Information files.]
Please confirm at this time whether or not your submission contains all raw data required to replicate the results of your study. Authors must share the "minimal data set" for their submission. PLOS defines the minimal data set to consist of the data required to replicate all study findings reported in the article, as well as related metadata and methods (https://journals.plos.org/plosone/s/data-availability#loc-minimal-data-set-definition).
For example, authors should submit the following data:
- The values behind the means, standard deviations and other measures reported;
- The values used to build graphs;
- The points extracted from images for analysis.
Authors do not need to submit their entire data set if only a portion of the data was used in the reported study.
If your submission does not contain these data, please either upload them as Supporting Information files or deposit them to a stable, public repository and provide us with the relevant URLs, DOIs, or accession numbers. For a list of recommended repositories, please see https://journals.plos.org/plosone/s/recommended-repositories.
If there are ethical or legal restrictions on sharing a de-identified data set, please explain them in detail (e.g., data contain potentially sensitive information, data are owned by a third-party organization, etc.) and who has imposed them (e.g., an ethics committee). Please also provide contact information for a data access committee, ethics committee, or other institutional body to which data requests may be sent. If data are owned by a third party, please indicate how others may request data access.

We confirm at this time our submission contains all raw data required to replicate the results of our study, https://github.com/extezo/plos-one.

We submit the following data:

- The values behind the means and standard deviations,

- The values used to build graphs,

- The points extracted from images for analysis.

The ethical restrictions on sharing a de-identified data set exist and are imposed by the HECS Human Ethics Committee University of Waikato (Contact point for enquiries Lois Vuursteen lois.vuursteen@waikato.ac.nz).

The explanation of ethical restrictions on sharing a de-identified data set presented in the open repository:

Data collected from the survey must be stored on university computers. The identities of participants are kept confidential and do not relate to their results. Results can be used only for research dissemination at conferences, journals, research thesis, and teaching resources. For the purposes presented above, data can be obtained by request at the email address anast.mozhaeva@gmail.com.

3. Your ethics statement should only appear in the Methods section of your manuscript. If your ethics statement is written in any section besides the Methods, please move it to the Methods section and delete it from any other section. Please ensure that your ethics statement is included in your manuscript, as the ethics statement entered into the online submission form will not be published alongside your manuscript.

We changed the name of the part from Procedure to Method, since previously there was no Method part. The ethics statement now appears in the Methods section.

Modified text, line 217:

HECS-20-64, HECS-20-58 where 21 December 2020 was the start, and 29 January 2021 was the end of the recruitment period for this study. Written informed consent was obtained from all participants.

4. We note that Figure 4 and 10 in your submission contain copyrighted images. All PLOS content is published under the Creative Commons Attribution License (CC BY 4.0), which means that the manuscript, images, and Supporting Information files will be freely available online, and any third party is permitted to access, download, copy, distribute, and use these materials in any way, even commercially, with proper attribution. For more information, see our copyright guidelines: http://journals.plos.org/plosone/s/licenses-and-copyright.

We require you to either (1) present written permission from the copyright holder to publish these figures specifically under the CC BY 4.0 license, or (2) remove the figures from your

submission:

a. You may seek permission from the original copyright holder of Figure 4 and 10 to publish the content specifically under the CC BY 4.0 license.

We recommend that you contact the original copyright holder with the Content Permission Form (http://journals.plos.org/plosone/s/file?id=7c09/content-permission-form.pdf) and the following text:
“I request permission for the open-access journal PLOS ONE to publish XXX under the Creative Commons Attribution License (CCAL) CC BY 4.0 (http://creativecommons.org/licenses/by/4.0/). Please be aware that this license allows unrestricted use and distribution, even commercially, by third parties. Please reply and provide explicit written permission to publish XXX under a CC BY license and complete the attached form.”

Please upload the completed Content Permission Form or other proof of granted permissions as an ""Other"" file with your submission.

In the figure caption of the copyrighted figure, please include the following text: “Reprinted from [ref] under a CC BY license, with permission from [name of publisher], original copyright [original copyright year].”

b. If you are unable to obtain permission from the original copyright holder to publish these figures under the CC BY 4.0 license or if the copyright holder’s requirements are incompatible with the CC BY 4.0 license, please either i) remove the figure or ii) supply a replacement figure that complies with the CC BY 4.0 license. Please check copyright information on all replacement figures and update the figure caption with source information. If applicable, please specify in the figure caption text when a figure is similar but not identical to the original image and is therefore for illustrative purposes only.

We have replaced the images in both cases.

5. Please include a copy of Table 2 which you refer to in your text on page 10.

Our apologies, this was a typographical error.

Modified text, line 308:

We report the coefficients of the defined approximation polynomials on our open repository.

6. Please review your reference list to ensure that it is complete and correct. If you have cited papers that have been retracted, please include the rationale for doing so in the manuscript text, or remove these references and replace them with relevant current references. Any changes to the reference list should be mentioned in the rebuttal letter that accompanies your revised manuscript. If you need to cite a retracted article, indicate the article’s retracted status in the References list and also include a citation and full reference for the retraction notice.

We have revised the references.

Modified text::

References

1. Cisco Visual Networking Index. Global - 2020 Forecast Highlights; 2020 [cited 2024 March 24]. Available from: https://www.cisco.com/c/dam/m/en_us/solutions/service-provider/vni-forecast-highlights/pdf/Global_2020_Forecast_Highlights.pdf.

4. Mantiuk RK, Denes G, Chapiro A, Kaplanyan A, Rufo G, Bachy R, et al. FovVideoVDP: a visible difference predictor for wide field-of-view video. ACM Transactions on Graphics. 2021;40(4):1–19.

6. Soundararajan R, Bovik AC. Video quality assessment by reduced reference spatio-temporal entropic differencing. IEEE Transactions on Circuits and Systems for Video Technology. 2013;23(4):684–694.

7. Kulikowski JJ. Some stimulus parameters affecting spatial and temporal resolution in human vision. Vision Research. 1971;11(1):83–93. March 11, 2024 13/15

8. Watson A, Ahumada A. The pyramid of visibility. Journal of Vision. 2016;16(12):567.

9. Luizov AV. Inertia of vision. Moscow, Russia: Oborongiz; 1961.

13. Lange HD. Research into the dynamic nature of the human fovea-cortex systems with intermittent and modulated light. The Journal of the Optical Society of America. 1958;48(11):784–789.

16. Campbell F, Cooper G, Robson J. Application of Fourier analysis to the visibility of gratings. The Journal of Physiology. 1968;197(3):551–566.

18. Watson A, Ahumada A, Farrell J. Window of visibility: psychophysical theory of fidelity in time-sampled visual motion displays. The Journal of the Optical Society of America. 1986;3(3):300–307.

20. Mantiuk R, Kim M, Ashraf M, Xu Q, Luo M, Martinovic J, et al. Practical Color Contrast Sensitivity Functions for Luminance Levels up to 10000 cd/m2. Color and Imaging Conference. 2020;28(1):1–6.

21. Laird J, Rosen M, Pelz J, Montag E, Daly S. Spatio-velocity CSF as a function of retinal velocity using unstabilized stimuli. Human Vision and Electronic Imaging. 2006;6057.

22. Mantiuk R, Ashraf M, Chapiro A. stelaCSF: a unified model of contrast sensitivity as the function of spatio-temporal frequency, eccentricity, luminance and area. ACM Transactions on Graphics. 2022;41(4):1–16.

28. Hubel DH. Eye, brain, and vision. Moscow, Russia: Mir; 1990.

29. International Telecommunications Union. Recommendation ITU-R BT. 709-6; 2015 [cited 2024 March 24]. Available from: https://www.itu.int/dms_pubrec/itu-r/rec/bt/R-REC-BT.709-6-201506-I!!PDF-E.pdf.

30. Fairchild MD. Color Appearance Models. Hoboken, NJ: John Wiley & Sons; 2013.

32. International Telecommunications Union. Recommendation ITU-R BT. 500-15; 2023 [cited 2024 March 24]. Available from: https://www.itu.int/dms_pubrec/itu-r/rec/bt/R-REC-BT.500-15-202305-I!!PDF-E.pdf.

33. Robson JG. Spatial and temporal contrast sensitivity functions of the visual system. Journal of the Optical Society of America. 1966;56(8):1141–1142.

34. Bampis CG, Li Z, Moorthy AK, Katsavounidis I, Aaron A, Bovik AC. Study of Temporal Effects on Subjective Video Quality of Experience. IEEE Transactions on Image Processing. 2017;26(11):5217–5231.

35. Bampis CG, Li Z, Moorthy AK, Katsavounidis I, Aaron A, Bovik AC. LIVE Netflix Video Quality of Experience Database; 2016 [cited 2024 March 24]. Available from: https://live.ece.utexas.edu/research/LIVE_NFLXStudy/nflx_index.html.

36. Lin JY, Song R, Wu CH, Liu TJ, and Wang, Kuo CCJ. MCL-V: A streaming video quality assessment database. The Journal of Visual Communication and Image Representation. 2015;30:1–9.

38. Davis J, Hsieh YH, Lee HC. Humans perceive flicker artefacts at as high as 500 H. Scientific reports. 2015;5(1):1–4.

Reviewer #1:

The authors created a setup for evaluating the detection threshold of spatio-temporal artefacts in movies and developed a mathematical model to predict those threshold. Furthermore, they conducted a comprehensive study to collect data, which in turn was used to test the model. Finally, the results were compared to existing models. This is really an impressive piece of work and I would like to congratulate the authors to it!

We thank the reviewer for the positive appraisal of our work.

Accordingly, I only have some minor comments:

line 73: reference Cecchi [2018] is missing

Modified text, line 72:

(More recent definitions of early vision, for example, that given by Cecchi [11], include computation of basic properties like shape and color.)

line 118 - 155: These paragraph my better fit into the discussion section.

The paragraph moved to the discussion section.

line 185: Why were especially these brightness values chosen?

Modified text, line 148:

We use a linear grid of average screen brightness values, which is suitable for further analysis with existing visibility models [22]. The minimum and maximum average brightness values used ensure that the test pattern is not distorted by the hard limits of the eight-bit brightness value limits.

Modified text in Discussion, beginning on line 413:

During the experiment, we considered increasing the number of brightness points, but the degree of nonlinearity was not adequately large enough to require more.

line 335: Table reference missing (??)

Modified text, line 308:

We report the coefficients of the defined approximation polynomials on our open repository.

Reviewer #2:

The summary of the research and overall impression is good, however, there is a lot of scope for improvement in the article for a better understanding of the reader. The strength of the article is the success in creating the model as expected, somewhere the methodology has been made complex by connecting it to the introduction part. This part has to be reworked majorly in the current article.

The authors have made a good start in the abstract and introduction part of the article, however, in comparing the research question to the previously published article, the results of the current study have also been discussed.

Thank you for your comment.

We reworked the methodology and introduction part in response to the suggestions made by both reviewers.

Figure 1 to 5 requires citation and repositioning in the text of the article.

Figure 1 repositioned to Model comparison part as Figure 9, second paragraph.

Modified text, label of, now, Figure 9:

Visualization of contrast sensitivity models (from left to right): Pyramid of visibility [8], FovVideoVDP [4], stelaCSF [22], Visibility model.

Figure 2 is repositioned as Figure 1.

Modified text, label of, now, Figure 1:

The stimulus used to generate Mira. The spectrum of the test signal, Normalized frequency is the digital frequency of the spectrum. The

horizontal dashed line represents the spatial sensitivity threshold [26].

Figure 3 is repositioned as, Figure 2.

Modified text, label of, now, Figure 2:

Structural diagram of the display system installation for research [26].

Figure 4 is repositioned as Figure 3.

Figure 5 is repositioned as Figure 4.

Modified text, line 184:

Using the presented equipment, we measured the relationship between the value of the specified pixel brightness and the screen's actual brightness used in the experiment. Fig 4 shows how it is possible to correspond the pixel brightness values used in the proposed work with the generally accepted brightness in candela per meter.

Line 67, figure 6 needs to be repositioned.

Figure 6 repositioned to Results part as Figure 5, first paragraph.

Line 75-80 require a reframing of the sentence for a better understanding of the reader.

Modified text, line 75:

The CSF is a bandpass filter that passes only visual stimuli that the observer can perceive. Only video artefacts in the passband region can be perceived by humans, hence the importance of CSF. Extant datasets on CSF have been collected with limitations. Datasets were collected firstly as a temporary measurement threshold [13]; secondly as the threshold for spatial contrast sensitivity only [14, 15]; and simultaneous spatial and temporal measurements using targets that were sinusoidal in both space and time [16 Today, CSF

research still does not present data sets with dependent spatial and temporal frequency measurements.

Line 117, the same word has been repeated?

Modified text, line 116:

Each participant requires about 30 minutes to perform 960 evaluations of various interactions of luminance, spatial, and temporal frequency values.

While the study appears to be good, I advise the authors to rework the methodology part of the study, giving better clarity for the current work being published. Needs clarification on what basis the sample size for both the 1st and 2nd experiments was decided.

Modified text, line 253:

All experiments continued until the confidence interval fell below 5% of the current mean value for each point across participants. Consequently, the basis of the sample size for the 1st and 2nd experiments was decided according to Rec. ITU-T Bt.500-15 standard [32].

The methodology followed for the comparison of the model needs an explanation of the procedure/methods.

We provided an explanation of the procedure followed for the comparison of the models.

Modified text, line 331:

All three models generate time coefficients that change with the frame, which are then passed to the VQM to calculate the score value. We averaged quality predictions across all frames in each video sequence in the data sets. We fit a non-linear model to each metric that maps from predictions of the average human scores in the open datasets. We report the prediction results as the Pearson correlation coefficient (PLCC). We also report the average of PLCCs computed per dataset, including upper and lower bounds.

Values excluded from the study are missing in the result part, although has been discussed in the discussion part with justification.

Modified text, line 288:

It should also be noted that participants noticed flickering at the higher frequencies tested at 15, 40 Hz (pixel brightness level is 200), at 36 Hz (pixel brightness level is 160), 13, 20 and 48 Hz (pixel brightness level is 80); 40 and 46 Hz (pixel brightness level is 40). These values were reasonably excluded from the analysis; details are discussed below.

Modified text, line 428:

Based on the exact repeatability of the frequencies of these anomalies, we assumed that the recorded effect is associated more with the test equipment's imperfections than with the visual system's properties. We did additional measurements using a photosensor and an oscilloscope, but no spurious frequencies were detected in the generated stimulus. Also, it must be considered that at these frequencies, the contrast sensitivity of vision becomes commensurate with the value of the quantization noise of the display device and the PWM backlight controller, as well as with the sampling parameters of the measuring equipment. We excluded these changes when processing the results and provided the experimental data for further research.

Points in lines 295-297 can be a part of the discussion.

Thank you for the suggestion, but in our opinion, it would be more understandable for readers if we presented this information in the results section.

Line 363, is that Table 1? In the content, it has been written as table 2, but there is no table 2 found in the present article.

Modified text, line 308:

We report the coefficients of the defined approximation polynomials on our open repository.

Table 1, representation of data in comparison to other models, PLCC need to be written as Pearson Linear Correlation Coefficient.

We have written PLCC as Pearson Linear Correlation Coefficient in Table.

Discussion can be elaborated, by moving some of the points from the previous parts of the article. Authors should have a comparison of the current model with the previous ones in the discussion part as this is not one of the main objectives as per the title.

Following the first reviewer's comments, we elaborated on the Discussion part. The two last paragraphs from Related Work were moved to the discussion. Also, following your suggestion, a comparison of the current model with the previous models was moved to the Discussion part, including Fig. 9, Fig. 10 and associated exposition in the text.

Limitations of the study can be discussed.

We added information on the Limitations of the study in the Discussion part.

Modified text, line 449:

Limitations of the experiment include imperfections in the test apparatus and the measurement process. Due to the limitation of contrast and maximum screen pixel brightness, the characteristics of vision in the state of its full adaptation were studied. Changes in the mode of a non-adapted HVS, which can be used, for example, when observing high-contrast scenes in HDR systems, require an appropriate stimulus generation device.

Full data sheet access has not been attached to the article.

Our submission contains all raw data required to replicate the results of our study, https://github.com/extezo/plos-one:

- The values behind the means and standard deviations,
- The values used to build graphs,
- The points extracted from images for analysis,
- The coefficients of the defined approximation polynomials of s(k, f, 120) derivatives.

The reference style has to be reworked as there is a discrepancy in the list of references.

The changes to the references are presented above in response to the journal requirements.
